# Validity of National Institutes of Health Stroke Scale for Severity of Stroke to Predict Mortality Among Patients Presenting With Symptoms of Stroke

**DOI:** 10.7759/cureus.10255

**Published:** 2020-09-05

**Authors:** Umar Farooque, Ashok Kumar Lohano, Ashok Kumar, Sundas Karimi, Farah Yasmin, Vijaya Chaitanya Bollampally, Margil R Ranpariya

**Affiliations:** 1 Neurology, Dow University of Health Sciences, Karachi, PAK; 2 Medicine, Peoples University of Medical and Health Sciences for Women, Nawabshah, PAK; 3 Internal Medicine, Peoples University of Medical and Health Sciences for Women, Nawabshah, PAK; 4 General Surgery, Combined Military Hospital, Karachi, PAK; 5 Cardiology, Dow University of Health Sciences, Karachi, PAK; 6 Neurological Surgery, Capital Medical University, Beijing, CHN; 7 Internal Medicine, Surat Municipal Institute of Medical Education and Research, Surat, IND

**Keywords:** stroke, validity, health stroke scale system, mortality, humans, national institutes of health stroke scale

## Abstract

Introduction

Cerebrovascular accident (CVA), also termed as stroke, is the third leading cause of mortality and the most common cause of disability globally. The National Institutes of Health Stroke Scale (NIHSS) is a valid assessment tool utilized to determine the severity of the stroke and can be used to prioritize patients to design treatment plans, rehabilitation, and better clinical outcomes. The primary objective of this study was to determine the validity of the NIHSS to predict mortality among patients presenting with symptoms of a stroke.

Material and methods

This was a descriptive case-series conducted over a period of six months between September 2019 and February 2020 at a tertiary care hospital in Nawabshah, Pakistan. The sample population included 141 patients admitted within 24 hours of the onset of symptoms of a stroke. A neurological examination of the patients was performed. On admission, stroke severity was evaluated with the NIHSS. After an initial clinical evaluation, patients underwent a non-enhanced computed tomography (CT) scan of the brain. The score of NIHSS and mortality at 72 hours were recorded on the pre-defined proforma by the investigators. All statistical analysis was performed using Statistical Package for Social Sciences (SPSS) version 23.0 (Armonk, NY: IBM Corp).

Results

The mean age of the participants was 52.37±8.61 years. 68.1% of patients were hypertensive, 29.1% were diabetic, and 36.9% of patients were found with hyperlipidemia. The mortality rate was 41.1%. The mean NIHSS score was 16.68±6.72 points. The findings of this study demonstrated that the score of 14.9% cases was good (0-6 points), the score of 29.1% cases was moderate (7-15 points), and the score of 56% cases was poor (≥16 points). There was a significant association of NIHSS score with mortality (p<0.001).

Conclusions

Baseline NIHSS score has a profound association with mortality after acute stroke. It can help clinicians decide whether to provide thrombolytic treatment, rehabilitation or a combination of both in these patients and decrease the mortality rate. However, more studies are needed to potentiate these conclusions.

## Introduction

Stroke is a cerebrovascular disease defined as rapidly developing signs of the focal or global loss of cerebral function with clinical symptoms for a minimum of 24 hours or resulting in death, with no other cause except vascular origin. It is ranked as the third most common cause of mortality and the leading cause of long-term serious disability. According to the World Health Organization (WHO), nearly 15 million people suffer from stroke annually worldwide, with five million succumbing to this disease while another five million remaining severely disabled for the rest of their lives [1]. This chronic neurological disorder is estimated to result in 7.8 million deaths by 2030 with a major proportion in third world countries [2]. Nearly 70% of strokes and 87% of both stroke-related mortality and disability-adjusted life-years have been reported in low and middle-income countries with the incidence of stroke being doubled over the last four decades. The number of patients having stroke-related disability-adjusted life years in the South Asian countries is approximately seven-folds greater mainly owing to the abundance of vascular risk factors such as tobacco smoking, reduced physical activity, unhealthy diet, abdominal obesity, hypertension and diabetes coupled with lack of preventive strategies [3].

Stroke can be further subdivided into two types mainly, ischemic and hemorrhagic, depending on the disturbances of cerebral circulation. Ischemic stroke occurs due to the obstruction of a cerebral artery either due to an embolus or a thrombus resulting in ischemic in part or all of the territory supplied by the occluded artery. In contrast, hemorrhagic stroke is mainly due to arteriolar hypertensive diseases, rarely due to coagulation disorders, cerebral malformations, and diet [3]. Patients who suffer from transient ischemic attack (TIA) and minor ischemic stroke (MIS) have a higher risk of recurrent stroke and their identification is essential for better adherence to preventive strategies [4]. Clinical insight rules help decide patients in whom treatment should be prioritized and stroke recurrence can be a strong interpretation to enable clinicians to make clinical decisions [5]. Prediction scores help classify TIA into subgroups based on initial stroke risk, hence providing triage conclusiveness in primary and secondary care [6].

Hospital level performance of Medicare beneficiaries in the United States for cardiovascular diseases is now being monitored, which comprises 30-day mortality rates [7-10]. Acute ischemic stroke (AIS) has also been included in this, as on admission of the patient, stroke severity is a potent determinant of practical outcomes in AIS [7-9, 11-13]. The National Institutes of Health Stroke Scale (NIHSS) is a valid assessment tool for the initial severity of stroke on admission and helps to predict the mortality in AIS [13-15]. Prediction of mortality plays a prominent role to help stratify prognosis and clinical outcomes. It is a non-linear conventional scale highly suggestive of initial functional recovery and long-term clinical consequences. It can be easily performed by researchers, nurses, and physicians alike and has shown intra-rater and inter-rater reliability taking approximately five to eight minutes for completion. It comprises of 15 items with each having responses scored on a 0-4 points scale. The overall score ranges from 0-42 points with higher scores specifying pronounced neurological deficits [16-17]. It assesses multiple neurological characteristics of the patients ranging from the level of consciousness, speech and language, neglect, communication, the field of vision, eye movements, facial symmetry, motor strength, sensation, and coordination of the patients.

The evaluation of the neurological condition of ischemic stroke patients can direct clinicians regarding prognosis, management, and appropriate treatment in these subjects [18]. Stroke contributes to the health-care burden globally, and data regarding its prevalence and risk factors can help plan medical and social services [19]. Accurate early outcome prediction using NIHSS helps in providing prevention strategies and categorization of patients in designing treatment plans, rehabilitation, nursing facility care, and better outcomes [20]. Hence, the primary objective of this study was to determine the validity of the NIHSS in predicting mortality among patients presenting with symptoms of a stroke. A secondary aim was to determine the impact of socio-demographic and clinical factors on the NIHSS score among stroke patients. 

## Materials and methods

Study setting and design

This was a descriptive case-series conducted from September 2019 to February 2020 for a duration of six months at a tertiary care hospital in Nawabshah, Pakistan.

Sample size, exclusion, and inclusion criteria

A sample size of 141 patients was calculated using the Raosoft sample size calculator by taking prevalence of mortality due to stroke of 40% [21] at a confidence interval of 95%, and a 5% margin of error. All patients aged 15-70 years admitted within 24 hours of the onset of symptoms of stroke and having computed tomography (CT) scan brain plain for confirmation were included in this study. We excluded any patient with subarachnoid hemorrhage (SAH), subdural hematoma, space-occupying lesion, diabetes with complications, hypoglycemia, asymptomatic cerebrovascular accident (CVA)/TIA/onset of symptoms >24 hours, complete heart block, head injury, gross anemia, previous brain lesions including tuberculoma, meningitis, encephalitis or hydrocephalus, chronic renal failure/acute myocardial infarction, and those who did not give written consent for the study.

Sampling technique and data collection

A non-probability convenience-based sampling technique was employed to collect data. The patients were informed regarding the risks and benefits of the study and both written and verbal informed consent was taken from the participants. Baseline clinical investigations were done and neurological and physical examinations were performed on all patients. The neurological examination included the level of consciousness, asking for month and age, asking to blink eyes and squeeze hands, testing for horizontal extraocular movements, testing for the visual field, testing facial palsy, left motor arm drift, right motor arm drift, left motor leg drift, right motor leg drift, limb ataxia, taste sensation, testing aphasia, dysarthria, testing inattention/extinction. Stroke severity on admission was evaluated with the NIHSS through a pre-defined proforma. After an initial clinical evaluation, patients underwent a non-enhanced CT scan brain plain. Other variables such as age, gender, hypertension, diabetes mellitus, and admission number were also recorded. The NIHSS was considered as significant for a score of >7 associated with bad prognosis, increased risk of death, and severe disability, and for a score of <3 associated with good functional recovery (i.e. able to live independently with the return of social activities). The validity was assessed as follows: good score (if the score is between 0-6 points), moderate score (if the score is between 7-15 points), and poor score (if the score is ≥ 16 points).

Statistical analysis

All statistical analysis was performed using Statistical Package for Social Sciences (SPSS) version 23.0 (Armonk, NY: IBM Corp). Data were presented as mean and standard deviation for qualitative whereas frequencies and percentages were recorded for quantitative variables such as age, gender, hypertension, diabetes mellitus, hyperlipidemia, and clinical severity of deficit measured by NIHSS. The score of NIHSS and mortality at 72 hours were recorded and Chi-square was applied to evaluate the association between good/poor NIHSS score and mortality.

## Results

Socio-demographic and clinical characteristics of the participants

Out of a total of 141 participants, nearly three-quarters (n=101/141, 71.6%) were males while the rest (n=40/141, 28.4%) were females. The mean age of study participants was 52.37±8.61 years. More than half (n=82/141, 58.0%) of our participants were aged > 50 years. With regards to clinical factors, more than two-third (n=96/141, 68.1%) of the participants had hypertension while less than one-third (n=41/141, 29.1%) had diabetes mellitus. Additionally, nearly two-fifth (n=52/141, 36.9%) of the study participants had hyperlipidemia, as shown in Table 1.

**Table 1:** Demographic and clinical factors of acute ischemic stroke patients

Variables		N (%)
Gender	Male	101 (71.6%)
	Female	40 (28.4%)
Age (years)	≤50	59 (42.0%)
	>50	82 (58.0%)
Hypertension	Yes	96 (68.1%)
	No	45 (31.9%)
Diabetes Mellitus	Yes	41 (29.1%)
	No	100 (70.9%)
Hyperlipidemia	Yes	52 (36.9%)
	No	89 (63.1%)

Mortality rate and NIHSS score of the participants

**Figure 1:**
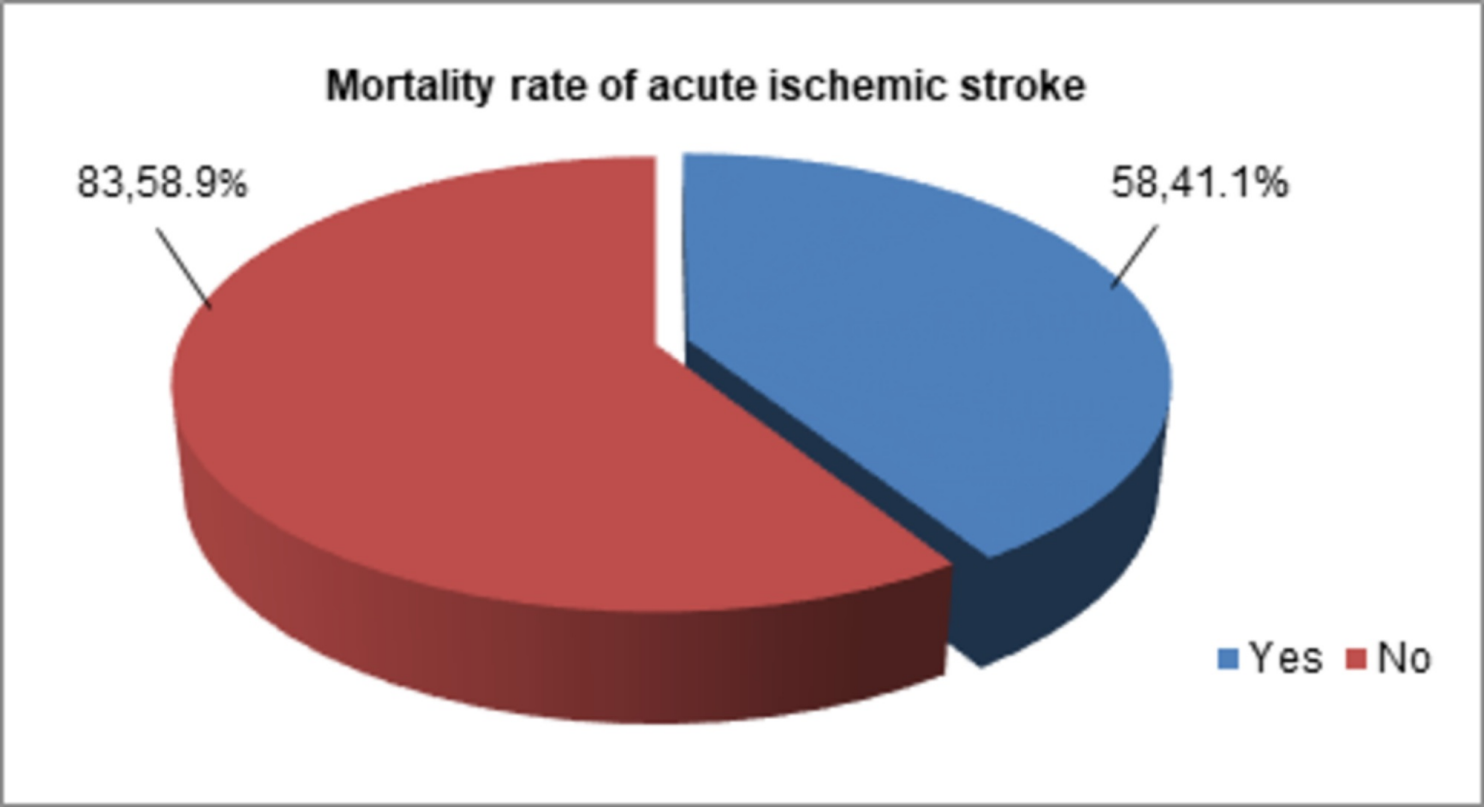
Mortality rate of acute ischemic stroke patients

Mortality was observed in nearly two-fifth (n=58/141, 41.1%) of stroke patients in this study, as demonstrated in Figure 1.

The mean NIHSS score of the study participants was 16.68±6.72 points. The descriptive statistics of examination by NIHSS score are presented in Table 2.

**Table 2:** Descriptive statistics of examination by NIHSS score NIHSS: National Institutes of Health Stroke Scale

	NIHSS variables	Mean ± standard deviation
1	Level of consciousness	1.31±0.96
2	Ask month and age	0.81±0.71
3	Blink eyes and squeeze hands	0.96±0.75
4	Test horizontal extraocular movements	0.88±0.75
5	Test visual fields	1.14±0.89
6	Test facial palsy	1.19±0.95
7	Test left arm motor drift	1.47±1.37
8	Test right arm motor drift	1.59±1.41
9	Test left leg motor drift	1.26±1.13
10	Test right leg motor drift	1.22±1.13
11	Test limb ataxia	0.82±0.69
12	Test sensation	0.75±0.72
13	Test language/aphasia	1.18±0.99
14	Test dysarthria	0.96±0.71
15	Test extinction/inattention	1.01±0.74

**Figure 2:**
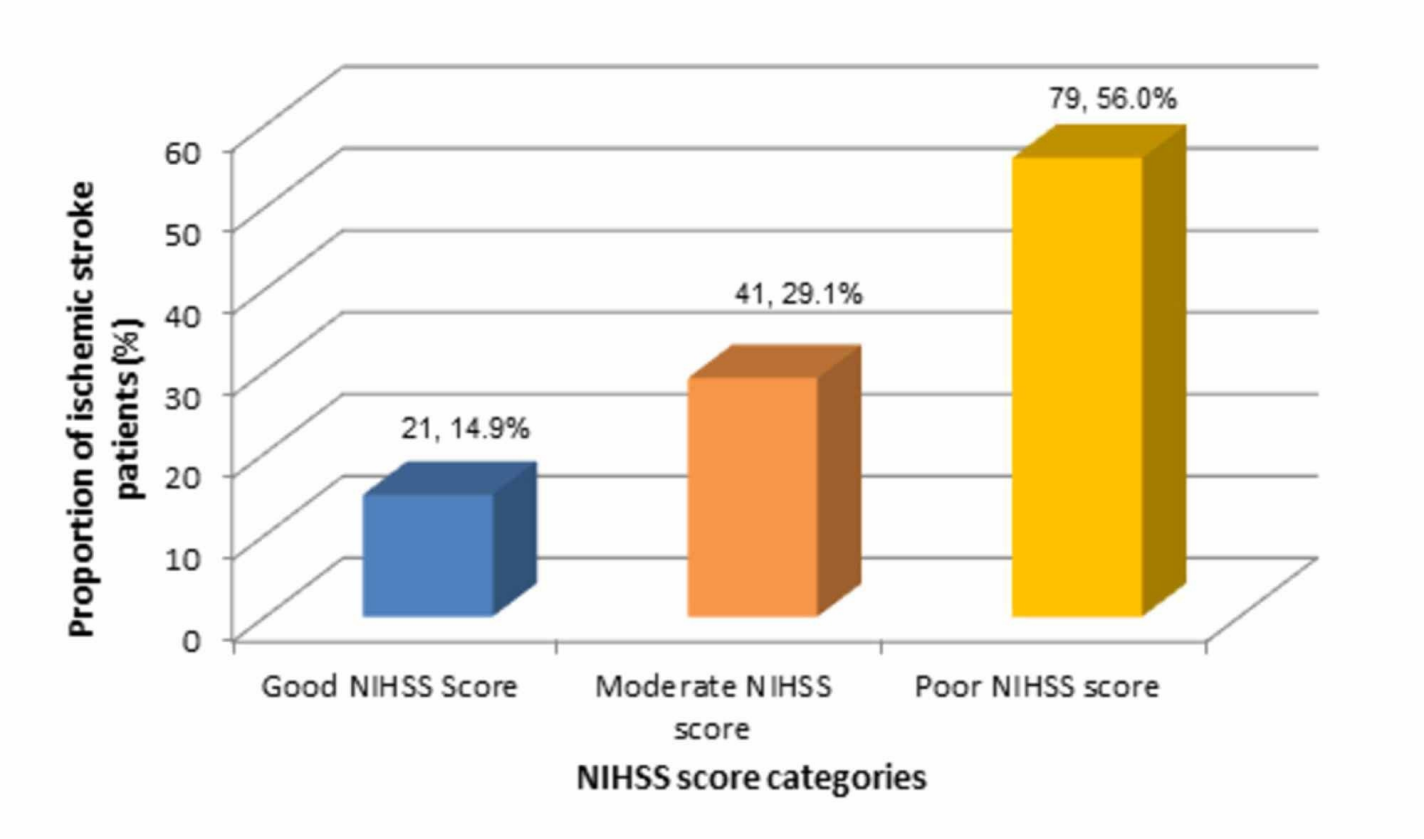
Distribution of acute ischemic stroke patients according to NIHSS categories NIHSS: National Institutes of Health Stroke Scale

The highest proportion (n=79/141, 56%) of stroke patients in this study reported poor NIHSS score, followed by less than one-third (n=41/141, 29.1%) with moderate score and the least proportion (n=21/141, 14.9%) reported good NIHSS score, as shown in Figure 2.

The descriptive statistics of NIHSS according to different categories is presented in Table 3.

**Table 3:** Descriptive statistics of score on the NIHSS according to different score categories NIHSS: National Institutes of Health Stroke Scale

	Good (0-6) (N=21)	Moderate (7-15) (N=41)	Poor (≥16) (N=79)
Mean ± standard deviation	5.28±0.71	12.48±2.52	21.89±2.43
95% confidence interval	4.95-5.61	11.69-13.28	21.35-22.44
Median (interquartile range)	5.00 (1)	13.00 (1.50)	22.00 (3)
Range	2	9	12
Minimum	4	6	18
Maximum	6	15	30

Associations of NIHSS score with mortality, demographic and clinical factors

Mortality was significantly associated with NIHSS score categories (p<0.001), as shown in Table 4. There was no mortality in stroke patients with good NIHSS score (n=21/21, 100%) while mortality was observed in a minor proportion (n=4/41, 9.8%) of participants with moderate NIHSS score. In contrast, mortality was observed in the highest proportion (n=54/79, 68.3%) of stroke patients with poor NIHSS score. Most of the participants with good NIHSS score (n=13/21, 61.9%), moderate NIHSS score (n=31/41, 75.6%) and poor NIHSS score (n=57/79, 72.2%) were males in comparison to females, however this difference did not reach statistical significance (p=0.520). More than half of the participants (n=12/21, 57.1%) with good NIHSS score were aged >50 years while more than one-third (n=15/41, 36.6%) of those with moderate NIHSS score were aged ≤50 years, and a higher proportion (n= 44/79, 55.7%) of participants with poor NIHSS score were aged >50 years, this difference again was not statistically significant (p=0.715). A higher proportion of participants with good NIHSS score (n=14/21, 66.7%), moderate NIHSS score (n=30/41, 73.2%), and poor NIHSS score (n=52/79, 65.8%) belonged to the hypertensive group (p=0.707). Furthermore, there was no significant association of NIHSS score with diabetes mellitus (p=0.435) and hyperlipidemia (p=0.493).

**Table 4:** Associations of NIHSS with mortality, demographic and clinical factors NIHSS: National Institutes of Health Stroke Scale

Variables		NIHSS			p-value
		Good score (0-6 points) N=21	Moderate score (7-15 points) N=41	Poor score (≥16 points) N=79	
Mortality	Yes	0 (0%)	4 (9.8%)	54 (68.3%)	<0.001*
	No	21 (100%)	37 (90.2%)	25 (31.6%)	
Gender	Male	13 (61.9%)	31 (75.6%)	57 (72.2%)	0.520
	Female	8 (38.1%)	10 (24.4%)	22 (27.8%)	
Age	≤50 years	9 (42.9%)	15 (36.6%)	35 (44.3%)	0.715
	>50 years	12 (57.1%)	26 (63.4%)	44 (55.7%)	
Hypertension	Yes	14 (66.7%)	30 (73.2%)	52 (65.8%)	0.707
	No	7 (33.3%)	11 (26.8%)	27 (34.2%)	
Diabetes mellitus	Yes	6 (28.6%)	15 (36.6%)	20 (25.3%)	0.435
	No	15 (71.4%)	26 (63.4%)	59 (74.7%)	
Hyperlipidemia	Yes	8 (38.1%)	18 (43.9%)	26 (32.9%)	0.493
	No	13 (61.9%)	23 (56.1%)	53 (67.1%)	

## Discussion

NIHSS is a valid assessment tool utilized to predict multiple stroke functional outcomes [12, 16]. NIHSS can be utilized to assess stroke severity that is a powerful predictor of mortality following AIS [13-15]. A study conducted in Taiwan comprising 360 hospitalized patients with AIS at the time of hospital admission after three months’ duration of stroke demonstrated NIHSS to be a strong predictor of mortality with an odds ratio of 1.17 (95% CI, 1.12-1.22) [22]. Another Swiss study on a sample size of 479 patients found older age and a higher NIHSS score to be the only predictors of 30-days mortality after stroke [15]. A German study reported NIHSS score to be a prominent predictor of 100-days survival within the initial six hours of hospital admission [23]. A study conducted by Get With The Guidelines (GWTG)-Stroke reported NIHSS to be a strong variable in predicting in-hospital mortality (c-statistic increased from 0.72 to 0.85) [24].

In our study, the mean age of AIS subjects was 52.37±8.61 years with more than half (58%) of the patients above 50 years of age. Another study reported a large proportion (86%) of the participants to be >45 years of age with an average of 64 years [25]. Shabbir and his colleagues in their study demonstrated stroke to be a disease of the geriatric population [26]. Elderly age has been reported to demonstrate higher baseline NIHSS score (> 16 points) and a poor outcome on Glasgow coma scale (a score of 3-5 points) while younger age is associated with low NIHSS score (a score of 0-6 points) at admission and a better outcome (a score of 1-2 points on Glasgow coma scale) [25]. There was an equal distribution of males and females in a local study [25]. In contrast, Khan and his colleagues reported a male to female ratio of 1:05:1 [19]. In our study, the male-to-female ratio was found to be comparative with these studies.

Fayyaz and his colleagues conducted a study on a cohort of 132 patients to determine clinical outcomes in diabetic patients suffering from ischemic stroke. According to the findings of the study, good recovery was displayed in diabetic and non-diabetic patients who were less than 40 years of age. A higher mortality rate was observed in the diabetic subgroup aged > 40 years and ≥60 years in comparison to non-diabetic patients [27]. In our survey, diabetes was reported as a comorbidity in 29.1% of ischemic stroke subjects. A study reported the mean baseline NIHSS score to be 18.20 (2-39) points. Nearly three-fifth of the patients (59.3%) had a baseline NIHSS score of > 16 points and displayed severe neurological deficit on hospital admission, and poorer clinical outcomes on the seventh day of admission as measured by the Glasgow outcome scale [25]. Our study showed that the mean score of NIHSS in the study patients was 16.68±6.72 with the lowest score being four and the highest being 30. In our study, it was observed that those who had a score of > 16 had a higher mortality rate, i.e. 68.3%.

Adams et al. carried out a study to compare baselines NIHSS score and the Trial of ORG 10172 in Acute Stroke Treatment (TOAST) score among 1,281 ischemic stroke patients to predict clinical outcomes at seven days and three months post-stroke using the Barthel index and the Glasgow outcome scale. An excellent outcome was defined as a score of 1 on the Glasgow outcome scale and a score of 19 or 20 using the Barthel index [11]. A similar study was conducted by Ahmed and his colleagues to assess neurological impairment using the NIHSS and functional outcomes on the seventh day of admission using the Barthel index. NIHSS and Barthel index score ranged from 2-28 points and 0-80 points, respectively [28]. The findings of a study conducted by Liu X et al. in Northwest China to determine functional outcomes in ischemic stroke subjects concluded that 43.8% of the subjects had poorer outcomes with predictors being advanced age, previous stroke history, and a higher NIHSS score [29].

However, this study has a few limitations. Firstly, it was conducted at a single tertiary care center in Pakistan. Secondly, the sample population was small which limits the generalizability of the findings. Hence, more studies conducted at multiple primary care settings involving a larger sample size and a variety of ethnic populations are required to further potentiate the results of the current study.

## Conclusions

Patients presenting with acute ischemic stroke and neurological deficit should have NIHSS be applied to them as the findings of this study concluded that NIHSS baseline score has a strong positive correlation with mortality and also helps clinicians decide whether to provide thrombolytic treatment, rehabilitation or a combination of both in these patients. This can lead to a reduced mortality rate. Further large-scale analysis is needed to validate the results of this study.
